# Colistin Sensitivity and Factor H-Binding Protein Expression among Commensal *Neisseria* Species

**DOI:** 10.1128/mSphere.00175-21

**Published:** 2021-06-16

**Authors:** Stephen A. Clark, Steve Gray, Adam Finn, Ray Borrow

**Affiliations:** a Meningococcal Reference Unit (MRU), Public Health England (PHE), Manchester, United Kingdom; b School of Cellular and Molecular Medicine, University of Bristol, Bristol, United Kingdom; Baylor College of Medicine

**Keywords:** *Neisseria*, carriage, commensal, factor H-binding protein, vaccines

## Abstract

Many bacterial carriage studies utilize colistin-containing media to select for Neisseria meningitidis among the diverse human pharyngeal milieu. These studies commonly report the isolation of *Neisseria* commensal species, with carriage rates of around 1% or less typically observed. Here, we describe the isolation of N. cinerea and N. polysaccharea from pharyngeal swabs using nonselective agar and confirm they are unable to grow on colistin-containing media. We also demonstrated colistin sensitivity among archived *Neisseria* commensal strains, including *N. cinerea*, N. polysaccharea, N. mucosa, and N. subflava. The distribution of *lptA* among these strains indicated that, while the phosphoethanolamine (PEA) transferase encoded by this gene confers colistin resistance, other mechanisms may lead to reduced susceptibility in some *lptA-*deficient strains. The majority of the *N. cinerea* and N. polysaccharea isolates expressed medium to very high levels of factor H-binding protein (fHbp), an important meningococcal vaccine antigen. Sequence analysis showed that the commensal fHbp peptide variants were similar in sequence to fHbp variants typically observed among invasive meningococci. Altogether, these results not only suggest that *Neisseria* commensal strains could be carried at much higher rates than previously reported but also raise questions about the impact of protein-based meningococcal vaccines on these unencapsulated commensals.

**IMPORTANCE** This study highlights the need for further work to accurately determine the pharyngeal carriage prevalence of *Neisseria* commensal bacteria (e.g., *N. cinerea* and N. polysaccharea) among the general population. Previous studies have clearly demonstrated the suppressive effect these commensal species can have on meningococcal colonization, and so the carriage prevalence of these species could be an important factor in the spread of meningococci through the population. Furthermore, the surface expression of the meningococcal vaccine antigen factor H-binding protein by many of these commensal strains could have important implications for the use of fHbp-containing vaccines. Carriage of these commensal species may influence the immune response to these vaccines, or conversely, the immune response elicited by vaccination may induce clearance of these potentially important members of the pharyngeal niche.

## INTRODUCTION

The bacterial genus *Neisseria* contains several species that naturally inhabit the human pharyngeal tract. Neisseria meningitidis is the most studied of these species due to its ability to cause devastating invasive disease including septicemia and/or meningitis. Carriage of N. meningitidis is a prerequisite for disease, and so the rate of meningococcal carriage within the population can be an important indicator when predicting the risk of invasive disease (1). For this reason, pharyngeal carriage surveys have been performed in many diverse settings and populations to determine the meningococcal carriage rate and to characterize the strains carried. In order to isolate N. meningitidis from the complex pharyngeal flora, selective culture media containing antibiotics such as vancomycin, nystatin, amphotericin, trimethoprim, and/or colistin (polymyxin E) are typically used (2–4). Commensal *Neisseria* species, such as N. lactamica, N. cinerea, N. polysaccharea, N. bergeri, and N. subflava are occasionally isolated on such media during these studies. Carriage rates for N. lactamica can be substantial and vary widely according to age, with carriage rates up to ∼40% in some younger age groups and much lower rates of <10% commonly observed in adolescents and adults (3–8). In contrast, carriage of other commensal *Neisseria* species are observed relatively rarely with rates typically reported at ∼1% or less (4, 8, 9).

Due to the low isolation rates and minimal clinical importance of these seemingly rare species, few studies have focused on them. In the last 10 years, however, the development of protein-based meningococcal vaccines has led to an interest in the distribution of vaccine antigens among these *Neisseria* commensals. In 2013, Muzzi and colleagues found genes encoding key meningococcal antigens, including factor H-binding protein (fHbp), a primary component of two licensed meningococcal vaccines, among *Neisseria* commensal strains (10). They reported that, while none of the eight N. lactamica strains harbored *fHbp*, all five *N. cinerea* strains and all seven of the N. polysaccharea strains studied possessed the gene. While none of the fHbp variants harbored by the commensals were identical to those within the two vaccines, antibodies against individual fHbp variants are broadly cross-reactive and will bind similar variants within the same fHbp subfamily or variant group (11). In 2017, Lavender et al. reported that *N. cinerea* can express functional fHbp with the ability to induce serum resistance by binding to human fH (12). Furthermore, antibodies raised in mice against an fHbp variant used in a licensed meningococcal vaccine (peptide variant 1) had substantial bactericidal activity against the fHbp-expressing *N. cinerea* strain (12).

In April 2016, a group B meningococcal disease outbreak began among adolescent students in South West England. The protracted outbreak, featuring four cases with two fatalities, persisted into the late summer of 2017 and concluded with the vaccination and prophylaxis of 138 individuals identified as part of an extended friendship group at high risk (13). Prior to vaccination or prophylaxis, 129 of these contacts consented to providing pharyngeal swabs to assess meningococcal carriage among the group. Meningococcal carriage was assessed by attempted isolation of N. meningitidis from the swabs using gonococcal (GC) selective agar comprising basal medium supplemented with 5% lysed horse blood with vancomycin, colistin, amphotericin, and trimethoprim (GC VCAT) (Oxoid, UK) and using real-time PCR assays targeting *sodC* and *ctrA*. To provide further detection and strain characterization, the swabs were also tested using porin A (PorA) and factor H-binding protein PCR-sequencing assays. Overall, the meningococcal carriage rate, as determined by culture, real-time PCR, and *porA* sequencing, was 32.6%. Interestingly, however, a much higher proportion of the swabs (78.3%) yielded a sequencable *fHbp* PCR product. The *fHbp* primers used in this assay bind to regions within flanking genes that are conserved among *Neisseria* species other than N. meningitidis (14). It was suspected, therefore, that many of the *fHbp* PCR products sequenced among these swabs were likely to have been amplified from the DNA of *Neisseria* commensal strains that did not grow on the selective GC VCAT agar. This explanation would contradict the results of previous carriage surveys that indicate that fHbp-possessing *Neisseria* commensal strains (e.g., *N. cinerea* and N. polysaccharea) are rarely carried.

The most likely hindrance to neisserial growth on GC VCAT is a sensitivity to colistin (polymyxin E) as colistin sensitivity among *Neisseria* commensals has been documented previously (15–18). Colistin binds to the negatively charged lipid A component of lipooligosaccharide (LOS) leading to a destabilization of the bacterial membrane and subsequent cell lysis (19). Modification of lipid A phosphate groups at the 1′ and 4′ positions with phosphoethanolamine (PEA) has been shown to increase net LOS charge and attenuate or eliminate binding of colistin (and other polymyxins), resulting in reduced susceptibility or resistance, respectively (19–21). Among *Neisseria* species, such substitutions are due to the activity of a PEA transferase encoded by the *lptA* gene (also termed *eptA*) (22). *lptA* is found in among the vast majority of N. meningitidis strains; however, it is notably absent in a number of commensal strains (21).

In this study, we describe attempts to isolate these previously nongrowing *Neisseria* from the *fHbp*-positive pharyngeal swabs. The isolated organisms, as well as additional *Neisseria* commensal strains, underwent further characterization to confirm the basis of their colistin sensitivity and to investigate variant distribution and expression of fHbp.

## RESULTS

### Identification of organisms isolated from pharyngeal swabs.

Using nonselective Columbia blood agar (CBA), 37 Gram-negative, oxidase-positive diplococcal (GNDC) organisms were isolated from the 94 swabs screened. Of these, 10 were found to be *fHbp* positive and underwent whole-genome sequence analysis.

Seven of these isolates were identified as *N. cinerea*, and the remaining three were N. polysaccharea (Table 1). One *N. cinerea* strain harbored a “new” *rplF* allele without an existing PubMLST allele identifier (ID). Maximum likelihood analysis showed the new allele clustered together with other *N. cinerea rplF* alleles (data not shown).

**TABLE 1:** Characteristics relating to colistin tolerance among 10 commensal *Neisseria* strains isolated from pharyngeal swabs

Isolate ID	Swab no.^*a*^	Species	*lptA* status	Colistin-sulfate zone size (mm)^*b*^	GC VCAT growth?
M19 240434	56	Neisseria polysaccharea	Absent	18	No
M19 240435	16	Neisseria cinerea	Absent	17	No
M19 240436	50	Neisseria cinerea	Absent	17	No
M19 240437	58	Neisseria polysaccharea	Absent	19	No
M19 240438	80	Neisseria cinerea	Absent	19	No^*c*^
M19 240439	30	Neisseria polysaccharea	Absent	19	No
M19 240440	124	Neisseria cinerea	Absent	19	No
M19 240441	45	Neisseria cinerea	Absent	19	No
M19 240442	81	Neisseria cinerea	Absent	19	No
M19 240443	42	Neisseria cinerea	Absent	19	No

a According to Clark et al. (13).

b A meningococcal control strain exhibited no zone of inhibition.

c Microcolonies observed on plates at 48 h.

Three of the 10 swabs that yielded a commensal isolate in this study were meningococcal positive by culture and/or molecular methods in the previous study (13). Swab 30, from which isolate M19 240439 (N. polysaccharea) was grown, was *sodC* and *ctrA* PCR positive, harbored a sequencable PorA gene (P1.5,2,36-2) and yielded a group W meningococcal isolate (BRI017). Swab 58, from which isolate M19 240437 (N. polysaccharea) was grown, was *ctrA* PCR positive and yielded a sequencable PorA product (P1.5-1,10-1,36-2). Finally, swab 16, from which M19 240435 (*N. cinerea*) was grown, was *sodC* PCR positive in the previous study (13).

### Colistin sensitivity and *lptA* status of organisms isolated from pharyngeal swabs.

All 10 strains failed to exhibit substantial growth on GC VCAT; however, microcolonies were observed after 48-h incubation for one of the isolates (*N. cinerea* [Table 1]). The suspected susceptibility to colistin was confirmed by disk diffusion. The commensals exhibited zone of inhibition around the disks of 17 to 19 mm, confirming that colistin is the agent responsible for the suppression on the GC VCAT agar. A meningococcal control strain (EMGM1) was also tested and exhibited no zone of inhibition, confirming resistance to colistin. Whole-genome sequence analysis revealed that all 10 strains lacked the *lptA* gene (Table 1).

### fHbp status and surface expression of organisms isolated from pharyngeal swabs.

For 7 of the 10 isolates, the *fHbp* allele exactly matched the corresponding allele sequenced directly from the swab in the previous study (Table 2) (13). For one isolate (isolate M19 240442 from swab 81), the isolate and swab-derived alleles were different by one base; probably indicative of a point mutation or inaccuracy in the sequencing. In another instance (isolate M19 240439 from swab 30), the swab-derived sequence trace was inconclusive with several “double-peaks” suggesting the presence of DNA from more than one additional *fHbp*-harboring strain. This swab did indeed also yield a group W meningococcal isolate in the initial study (BRI017). The final discordant isolate (isolate M19 240443 from swab 42) possessed a substantially different *fHbp* allele (1532, subfamily A/variant 3) in relation to the swab (1542, subfamily B/variant 1); however, the sequence trace exhibited some background “double-peaks” that correlated well with the isolate ID (data not shown).

**TABLE 2:** Factor H-binding protein status and surface expression among 10 commensal *Neisseria* strains isolated from pharyngeal swabs

Isolate ID	Species	Swab *fHbp* allele	Isolate *fHbp* allele	Isolate fHbp peptide	Isolate fHbp variant	fHbp surface expression^*a*^			
						Background MFI	fHbp-specific MFI^*b*^	Signal/ background ratio	Expression level
M19 240434	Neisseria polysaccharea	1282	1282	1008	3	358	2,951	8	Medium
M19 240435	Neisseria cinerea	108	108	108	1	200	3,668	18	Medium
M19 240436	Neisseria cinerea	108	108	108	1	238	6,062	25	High
M19 240437	Neisseria polysaccharea	1539^*c*^	1539^*c*^	NA^*c*^	NA^*c*^	ND	ND	ND	ND
M19 240438	Neisseria cinerea	47	47	61	1	181	11,989	66	Very high
M19 240439	Neisseria polysaccharea	Mixed 546/13	1694	1310	1	233	701	3	Low
M19 240440	Neisseria cinerea	15	15	15	1	151	27,285	181	Very high
M19 240441	Neisseria cinerea	522	522	447	1	100	394	4	Low
M19 240442	Neisseria cinerea	971	1693	456	1	295	2,457	8	Medium
M19 240443	Neisseria cinerea	1542	1532	401	3	250	1,837	7	Medium

a ND, not determined.

b Meningococcal control strains PMB1135 and PMB1745 produced fHbp-specific MFIs of 1,495 and 5,605, respectively.

c The allele features premature stop codon due to frameshift. NA, not available.

Seven of the 10 isolates possessed an *fHbp* allele encoding subfamily B/variant group 1 fHbp peptides, including representatives from both species (Table 2). Two of the remaining three isolates (one *N. cinerea* and one N. polysaccharea) harbored subfamily A (variant group 3) fHbp variants. The final strain (N. polysaccharea) possessed *fHbp* allele 1539, which encodes a peptide featuring an internal stop codon preventing the expression of a full-length fHbp protein.

Surface expression of fHbp was detected for all nine of the strains with a full-length fHbp coding region (Table 2). Three of the seven *N. cinerea* strains expressed fHbp at levels considered “high” or “very high” with the flow cytometric signal intensity of 25 to 181 times that of the background level measured by the nonspecific isotype control. Another three of the *N. cinerea* isolates expressed fHbp at moderate levels with signal/background ratios ranging from 7:1 to 18:1. The remaining *N. cinerea* strain, harboring a subfamily B fHbp peptide (peptide 447), expressed low levels of fHbp at the surface (signal/background ratio of 4:1). For the two N. polysaccharea strains harboring *fHbp* genes encoding intact peptides, one showed a moderate level of expression (signal/background ratio of 8:1), while the other exhibited a signal/background ratio only slightly above the defined threshold for positive expression (peptide 1310). The N. polysaccharea strain with a truncated fHbp peptide was not tested for surface expression (Table 2).

### Assessment of historic *Neisseria* commensal panel.

The Meningococcal Reference Unit (MRU) isolate archive contains a number of nonmeningococcal organisms isolated from systemic and nonsystemic clinical samples in recent years. Ten of these strains were deemed of interest and had previously undergone whole-genome sequence analysis. In order to further explore the relationship between *lptA* status and the ability of *Neisseria* to grow on GC VCAT, these 10 diverse nonmeningococcal *Neisseria* isolates were analyzed (Table 3). Eight of the 10 strains lacked the *lptA* gene. The remaining two strains that harbored the gene were N. lactamica and N. polysaccharea. Both strains grew well on GC VCAT. Of the eight *lptA*-deficient strains, one strain grew on GC VCAT (*N. cinerea*), suggesting the presence of an alternate colistin resistance mechanism in this strain.

**TABLE 3:** Colistin sensitivity, *fHbp* status, and fHbp surface expression of historic MRU *Neisseria* commensals^*a*^

Isolate ID	Sample type	Species	*lptA* status	GC VCAT growth?	*fHbp* allele	fHbp peptide	fHbp subfamily/ variant	Background MFI	fHbp-specific MFI^*b*^	Signal/ background ratio	fHbp expression
M14 240642	Blood	*N. mucosa*	Absent	No^*c*^	Absent	Absent	Absent	ND	ND	ND	ND
M15 240769	Throat swab	*N. cinerea*	Absent	No^*c*^	647	555	A/3	275	1611	6	Medium
M15 240827	Blood	N. polysaccharea	Absent	No	840	685	A/3	102	511	5	Low
M15 240955	Throat swab	N. polysaccharea	Absent	No	673	570	A/3	154	1848	12	Medium
M16 240028	Throat swab	N. lactamica	736	Yes	Absent	Absent	Absent	ND	ND	ND	ND
M16 240038	Blood	*N. cinerea*	Absent	Yes	512	437	B/1	127	3050	24	Medium
M16 240183	Bronchial aspirate	N. polysaccharea	Absent	No	673	570	A/3	200	5211	26	High
M16 240285	Throat swab	N. polysaccharea	133	Yes	1747	1346	B/1	161	4042	25	High
M16 240660	ND	*N. cinerea*	Absent	No^*c*^	110	110	B/1	106	1071	10	Medium
M18 240317	High vaginal swab	N. polysaccharea	Absent	No	570	494	A/3	110	506	5	Low

a ND, not determined.

b Meningococcal control strains PMB1135 and PMB1745 produced fHbp-specific MFIs of 1,529 and 5,226, respectively.

c Microcolonies observed on plates at 48 h.

All but two isolates, the sole representatives of N. lactamica and *N. mucosa*, harbored *fHbp* alleles (Table 3). The majority of these (5/8) belonged to subfamily A/variant 3 and the remaining to subfamily B/variant 1. All eight *fHbp*-harboring strains exhibited detectable fHbp surface expression. Expression levels ranged from low (among two N. polysaccharea isolates) to high (both N. polysaccharea and *N. cinerea*). The two *fHbp* null strains were not tested for expression.

### *lptA* status and colistin sensitivity of carriage survey isolates.

To determine the potential bias of using GC VCAT in a carriage study. An analysis of strains recently isolated in the national UKMenCar4 study was performed (4). The UKMenCar4 study utilized GC VCAT as the selective medium, and so all strains would have been initially isolated from this medium.

Of the 1,675 UKMenCar4 *Neisseria* genomes on PubMLST, only 17 (1.01%) were found to be *lptA* deficient. Two of these strains were *N. cinerea*, and the remaining 15 were N. subflava. These 17 strains were the only representatives of *N. cinerea* and *N. subflava* in the entire data set, with carriage prevalence for these species at 0.12% and 0.90%, respectively.

These 17 isolates were tested for growth on GC VCAT (Table 4). After 48-h incubation, four *N. subflava* strains exhibited significant growth. No substantial growth was observed for any other isolates, although three other *N. subflava* strains yielded microcolonies on the plate at the 48-h time point. None of the 15 *N. subflava* strains harbored *fHbp*, while both *N. cinerea* isolates possessed variant group 1/subfamily B *fHbp* genes (Table 4).

**TABLE 4:** Colistin sensitivity and *fHbp* status of *lptA-*deficient UKMenCar4 *Neisseria* isolates

Isolate ID	Species	*lptA* status	GC VCAT growth	*fHbp* allele	fHbp peptide	fHbp subfamily/ variant
BR40103	*N. subflava*	Absent	Yes	Absent	Absent	Absent
BR40599	*N. subflava*	Absent	No^*a*^	Absent	Absent	Absent
BR40608	*N. subflava*	Absent	No^*a*^	Absent	Absent	Absent
BR40664	*N. subflava*	Absent	Yes	Absent	Absent	Absent
BR41470	*N. subflava*	Absent	Yes	Absent	Absent	Absent
BR41637	*N. subflava*	Absent	Yes	Absent	Absent	Absent
CA42438	*N. subflava*	Absent	No^*a*^	Absent	Absent	Absent
MT40035	*N. subflava*	Absent	No	Absent	Absent	Absent
OX40632	*N. cinerea*	Absent	No	47	61	B/1
OX41971	*N. subflava*	Absent	No	Absent	Absent	Absent
OX42005	*N. subflava*	Absent	No	Absent	Absent	Absent
OX42006	*N. subflava*	Absent	No	Absent	Absent	Absent
OX42049	*N. subflava*	Absent	No	Absent	Absent	Absent
OX42181	*N. cinerea*	Absent	No	546	456	B/1
PL40548	*N. subflava*	Absent	No	Absent	Absent	Absent
PL42224	*N. subflava*	Absent	No	Absent	Absent	Absent
ST41782	*N. subflava*	Absent	No	Absent	Absent	Absent

a Microcolonies observed on plates at 48 h.

### fHbp sequence analysis and comparison.

To explore the possibility of cross-reactivity between vaccine-induced fHbp antibodies and fHbp variants expressed by *Neisseria* commensals, a comparison was performed between the commensal fHbp variants identified in this study and those possessed by both invasive and carried meningococci.

In this study, 19 commensal isolates harbored an *fHbp* allele encoding a full-length fHbp peptide (all *N. cinerea* or N. polysaccharea). Among these strains, 15 unique fHbp peptide variants were identified. Table 5 details the presence of these specific peptide variants among two panels of meningococcal isolates: invasive English and Welsh isolates within the Meningitis Research Foundation’s Meningococcus Genome Library (MRF-MGL) (*n* = 4,444) and the meningococcal isolates obtained as part of the UKMenCar4 carriage study (*n* = 1,420).

**TABLE 5:** Prevalence of commensal fHbp peptide variants among invasive and carried meningococcal data sets

Commensal fHbp peptide variant	Variant group/ subfamily	Commensal species (no. of isolates)^*a*^	Meningococcal data set			
			E&W^*b*^ MRF-MGL (invasive)		UKMenCar4 (carriage)	
			No. of strains	% prevalence	No. of strains	% prevalence
15	1/B	*N. cinerea* (1)	207	4.66	7	0.49
456	1/B	*N. cinerea* (2)	9	0.20	2	0.14
110	1/B	*N. cinerea* (1)	8	0.18	0	0.00
494	3/A	N. polysaccharea (1)	5	0.11	0	0.00
61	1/B	*N. cinerea* (2)	4	0.09	0	0.00
108	1/B	*N. cinerea* (2)	4	0.09	0	0.00
555	3/A	*N. cinerea* (1)	2	0.05	0	0.00
401	3/A	*N. cinerea* (1)	1	0.02	0	0.00
685	2/A	N. polysaccharea (1)	1	0.02	0	0.00
1008	3/A	N. polysaccharea (1)	1	0.02	0	0.00
437	1/B	*N. cinerea* (1)	0	0.00	0	0.00
447	1/B	*N. cinerea* (1)	0	0.00	0	0.00
570	3/A	N. polysaccharea (2)	0	0.00	0	0.00
1310	1/B	N. polysaccharea (1)	0	0.00	0	0.00
1346	3/A	N. polysaccharea (1)	0	0.00	0	0.00

a The commensal *Neisseria* species and the number of isolates in which the variant was identified in this study are shown.

b E&W, English and Welsh.

Of the 15 fHbp peptide variants identified in the commensal strains, only 2 were identified among meningococci isolates during UKMenCar4; 1.15 and 1.456, represented by seven and two meningococcal isolates, respectively.

Within the invasive English or Welsh MRF-MGL meningococcal data set, all but 5 of the 15 commensal fHbp peptide variants were identified. Variant 15 was represented by 4.7% of the invasive meningococcal isolates and was the sixth most common fHbp peptide within the data set. Each of the remaining nine variants present was represented by ≤0.2% of the invasive meningococcal strains (Table 5).

The five commensal fHbp peptide variants that were not identified among the invasive meningococcal strains were 437, 447, 570, 1,310, and 1,346. A wider search for these variants among all meningococcal records on PubMLST.org yielded two ST-11 strains from the United States with fHbp 447, and four diverse strains from various countries harboring fHbp 437.

**FIG 1:**
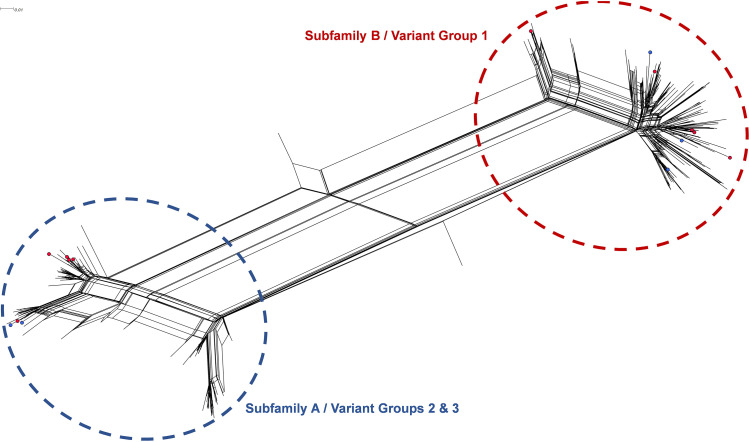
A NeighborNet split graph of 259 fHbp peptide sequences (excluding variable-length N-terminal linker, refer to Materials and Methods). The data set included all fHbp peptide variants harbored by English and Welsh meningococcal isolates in the MRF-MGL (*n* = 254), plus five variants observed only among the commensal strains described in this study. The 15 colored circles represent fHbp variants harbored by the commensal (*N. cinerea* and N. polysaccharea) strains. The 10 variants that were also seen among the invasive meningococci are colored red. The five fHbp peptide variants that were only seen among the *Neisseria* commensal isolates are colored blue.

The rarity of many of these commensal fHbp variants among meningococci suggests a degree of segregation in terms of the fHbp peptides harbored among the different species. To assess whether this segregation is reflected in the peptide sequence similarity of these variants, a phylogenetic analysis of the meningococcal and commensal fHbp peptide sequences was performed. The split graph in Fig. 1 illustrates the sequence similarity between all fHbp peptide variants within the English and Welsh (E&W) strains in the MRF-MGL, as well as the five additional variants found only among the commensals in this study (total *n* = 259). The 15 commensal fHbp variants (highlighted with colored circles) were distributed widely among the meningococcal variants within both subfamilies (Fig. 1). No notable clustering of the commensal variants was observed, indicating there is little sequence segregation between fHbp variants expressed by meningococci and those expressed by commensal strains.

## DISCUSSION

In this study, we attempted to isolate suspected *Neisseria* commensals from pharyngeal swabs collected following a meningococcal outbreak in the United Kingdom in 2017 (13). Following screening on nonselective blood agar, 10 *fHbp*-harboring *Neisseria* commensal strains (*N. cinerea* and N. polysaccharea) were isolated from 94 swabs tested. This low isolation rate reflects the difficulty in identifying and isolating target colonies among diverse flora using nonselective blood agar. Also, the viability of the organisms themselves could have been a limiting factor. During the initial study, only 18 out of 42 meningococcal PCR-positive swabs yielded a meningococcal isolate, and the swabs had subsequently undergone additional freeze-thaw cycles which could have impacted the recovery rate further (13). In the current study, the skim milk-tryptone-glucose-glycerol (STGG) broth was incubated in vancomycin solution prior to plating in order to suppress Gram-positive bacteria; however, the effectiveness of this technique was difficult to ascertain.

This study investigated colistin susceptibility among three distinct panels of *Neisseria* commensal strains. The results show a clear relationship between the presence of *lptA* and colistin tolerance, in accordance with the current understanding of PEA-mediated colistin resistance (19–21). Among *Neisseria* carriage isolates obtained during a recent United Kingdom carriage survey utilizing GC VCAT agar, 98.99% of the isolated strains harbored the *lptA* gene (4). Thirteen of the 17 strains that lacked the gene exhibited no or minimal growth on the GC VCAT agar upon retesting despite being originally isolated on the agar. The explanation for this contradiction is unclear, but we propose that it may be evidence of a yet unrecognized colistin suppression effect by cohabiting pharyngeal bacteria on the plate during the initial screening.

Of the 35 *lptA*-deficient strains analyzed in this study, 5 isolates (14.3%) were able to grow well on the GC VCAT. Also, for several of the remaining susceptible isolates, small microcolonies were observed on the agar, indicative of tolerant subpopulations. These findings suggest that alternative colistin resistance mechanisms are being utilized by a small proportion of these strains. A number of alternative mechanisms have been shown to confer colistin tolerance in other organisms (e.g., efflux pumps); however, further study of these strains is required in order to determine this mode of resistance (19).

Overall, these data suggest that the use of colistin-containing selective media (e.g., GC VCAT and modified Thayer-Martin) in carriage studies is likely to result in a substantial bias toward colistin-tolerant strains and, therefore, an underestimation of the prevalence of some nonmeningococcal *Neisseria* species (particularly *N. cinerea* and N. polysaccharea), as well as *Neisseria* carriage overall. Despite this, colistin-containing media is favored for most meningococcal carriage studies as it serves its primary purpose, namely, the isolation of N. meningitidis. Unfortunately, these studies represent the primary source of data on the prevalence of these lesser-studied commensal species (2, 4, 5, 23). Results of such studies typically report nonpathogenic *Neisseria* prevalence (excluding colistin-resistant N. lactamica) at ∼1% or less (4, 8, 9). In contrast, using the colistin-free LBVT.SNR medium, Knapp and Hook isolated *N. cinerea* from 28% of 202 American adults (24) and, using the same formulation, Nieto et al. reported 10% of *N. cinerea* among a small group of adults in Spain (18).

Culture-independent techniques have been utilized to assess the prevalence of *Neisseria* carriage to the genus level (reviewed in reference 25); however, only a few metagenomic studies have reported *Neisseria* carriage prevalence to the species level. In 2018, Fukui and colleagues detected *N. cinerea*-specific sequences among the tonsil bacteriome of 43% of a small group of adults from Japan (26). In 2011, Bogaert et al. used barcoded pyrosequencing to detect N. polysaccharea*-*specific sequences from 17% of nasopharygeal swabs taken from healthy 18-month-old Dutch children (27). The accuracy/reliability of identification of species using different culture-independent techniques is likely to vary, but these findings support the assertion that, in many populations, *Neisseria* carriage could be much higher than observed using colistin-containing culture media.

The availability of low-cost whole-genome sequencing in recent years has led to the characterization of previously unrecognized *Neisseria* species. In 2019, Diallo et al. described phylogenetic analyses of rarely isolated commensal *Neisseria* strains from several carriage surveys, the majority of which took place in Africa (28). They identified four putative novel *Neisseria* species (including *N. bergeri*) and three more groupings that may be prove to be additional species (subject to isolation of additional strains) (28). These findings indicate that the *Neisseria* genus is much more diverse than previously recognized. The relative rarity of these newly described species within isolate collections may simply reflect the low levels and limited geographic region in which they are carried (i.e., sub-Saharan Africa); however, it is conceivable that colistin sensitivity plays a key role in the likelihood of these strains being isolated and characterized. As such, there could yet be more *Neisseria* species to be identified.

The importance of obtaining accurate carriage prevalence data for nonpathogenic *Neisseria* is bolstered by evidence of important interactions between different *Neisseria* species in the pharyngeal niche. Data have emerged suggesting that *Neisseria* commensal species may be a source for antimicrobial resistance genes. In 2020, Chen and colleagues reported findings suggesting that over half of the quinolone-resistant meningococci isolated in Shanghai, China, acquired resistant *gyrA* genes from a small number of commensal *Neisseria* species, including *N. cinerea* (29). In addition to gene exchange, commensal *Neisseria* species can directly influence cohabiting meningococci in the pharyngeal tissues. The ability of N. lactamica carriage to restrict meningococcal colonization has been well described and forms the basis of research into using N. lactamica inoculation as a public health measure (30–32). Interestingly, N. lactamica is one of the few nonpathogenic *Neisseria* commensals to demonstrate consistent colistin tolerance, and so a comparatively higher carriage prevalence has been reported in previous studies for this species (3–8). This has made N. lactamica an obvious choice for studying interspecies interactions; however, other species such as *N. cinerea*, have also been implicated in directly influencing the progression or extent of meningococcal colonization (33).

Perhaps the most interesting aspect of the current findings is that many of the commensal strains studied possess and express fHbp and thus may influence immunity to or be a target for fHbp-containing vaccines. None of the N. lactamica, *N. mucosa*, or *N. subflava* strains in this study possessed *fHbp*; however, the majority of the *N. cinerea* and N. polysaccharea isolates harbored the antigen and exhibited substantial surface expression. To our knowledge, this study is the first demonstration of fHbp surface expression by N. polysaccharea, although a substantial proportion of the N. polysaccharea strains previously characterized possess frameshifted *fHbp* alleles, resulting in a lack of functional peptide expression (10). The methods used here to detect expression of fHbp have not been validated for commensal strains, but in relation to the mean fluorescence intensity (MFI) typically generated by meningococci, the results suggest moderate to very high levels of surface expression for the majority of these strains (34). It must be noted, however, that fHbp expression is subject to thermoregulation via the formation of an mRNA stem-loop preventing ribosome binding at lower temperatures (35). The *in vitro* techniques used to quantify fHbp in this study may not be reflective of those occurring during carriage at the lower temperatures of the pharyngeal tissues (34). In this study, modification of the standard meningococcal antigen surface expression assay (MEASURE) assay protocol was necessary due to the inability of these commensals to grow in GC broth. The fact that GC-based media may not provide for efficient growth of these species may perhaps be another hindrance to selection of these strains in carriage studies utilizing GC VCAT agar.

Many of the fHbp variants harbored by the commensal strains in this study were identified among N. meningitidis strains, albeit rarely. A phylogenetic analysis of the fHbp sequence variants found among the commensal strains and among invasive meningococci indicated little or no sequence segregation, with the commensal variants distributed widely among the meningococcal fHbp peptides. This finding raises the probability of cross-reactivity between vaccine-induced fHbp antibodies and fHbp expressed on the surface of *N. cinerea* and N. polysaccharea (and possibly other *Neisseria* commensals). This assertion is supported by data from Lavender and colleagues in 2017 (12) which demonstrated the serum bactericidal activity of anti-fHbp murine antibodies (raised against the fHbp vaccine variant 1.1) against *N. cinerea* expressing fHbp peptide 1.110. Interestingly, an *N. cinerea* strain expressing moderate amounts of this particular variant (1.110) was also identified among the historic panel in the current study (Table 3).

While we can demonstrate fHbp expression among commensal strains, predicting the possible impact of fHbp-containing vaccines on carriage is more difficult. The ability of fHbp-containing vaccines to elicit a reduction in carriage of invasive meningococci has yet to be conclusively demonstrated (36–38). The most recent data on this question come from a large-scale carriage study in Australian adolescents in 2017 (38). While Marshall et al. reported no reduction in the carriage of encapsulated meningococci following vaccination with a licensed fHbp-containing vaccine, a 29% risk reduction of carriage of unencapsulated meningococci was observed (38). It is therefore conceivable that protein-based group B vaccines could have a suppressive effect on other unencapsulated *Neisseria*, including fHbp-expressing commensal species.

To conclude, our findings suggest that the carriage of *Neisseria* commensals (*N. cinerea* and N. polysaccharea in particular) is likely to have been consistently underestimated in previous studies using colistin-containing screening media. Further investigation is required to accurately assess the true carriage prevalence of these commensals and the influence their presence could have on meningococcal colonization. The expression of fHbp (and conceivably other protein vaccine antigens) may make these species susceptible to clearance by antibodies raised against protein antigens within meningococcal vaccines. Future studies are required to assess the impact of any such changes in this important ecological niche.

## MATERIALS AND METHODS

### Selection of outbreak carriage swabs for *Neisseria* screening.

Pharyngeal swabbing of volunteers was performed as part of public health action in accordance with Public Health England guidelines; therefore, ethical approval was not required. Informed consent was obtained from all volunteers prior to pharyngeal swabbing (13). Among the 101 skim milk-tryptone-glucose-glycerol (STGG) swabs that yielded a sequencable *fHbp* product during the previous study, meningococcal isolates were grown from 18. Methods for the detection of *fHbp* from these swabs is detailed in the previous study (13). Only 7 of these 18 isolates harbored an *fHbp* allele that matched the allele sequenced directly from the swab. As these seven isolates showed no evidence of discrepancy, they were excluded from further analysis. The remaining 11 cultured swabs yielded an *fHbp* allele that differed from the allele harbored by the corresponding meningococcal isolate or showed mixed electropherogram traces (indicative of the presence of multiple allelic variants) and were therefore included in the analysis. All remaining 83 swabs were also screened regardless of meningococcal status (total *n* = 94).

### Isolation of suspected *Neisseria* commensals from swabs.

All swabs were stored at –80°C in STGG broth. Due to the failure of these *Neisseria* strains to grow on GC VCAT during the initial assessment, nonselective CBA (Oxoid, UK) was used. To improve the chances of isolation of suspected *Neisseria*, for each swab, 2 μl of STGG was added to 198 μl of vancomycin solution (6 μg/ml) and mixed. After 1 h at ambient temperature, all 200 μl of the inoculated solution was transferred to a CBA plate and spread using the lawn technique. CBA plates were incubated at 37°C with 5% CO_2_. At the 24- and 48-h time points, any colonies suspected to be *Neisseria* were subcultured on to a secondary CBA plate and incubated overnight. All subcultured organisms were tested using oxidase reagent, and all oxidase-positive isolates underwent Gram staining. All oxidase-positive Gram-negative diplococci were tested using the *fHbp* PCR sequencing assay, and *fHbp*-positive strains were stored for further characterization.

### Further characterization of fHbp-positive Gram-negative, oxidase-positive diplococci (GNDCs) from swabs.

The DNA of *fHbp*-positive organisms was extracted using MagMAX DNA Multi-Sample Ultra kit (ThermoFisher, UK) and whole-genome sequencing was performed using the Illumina HiSeq platform. Genomes were assembled using Velvet with optimal parameters defined by VelvetOptimiser (39). Assembled genomes were imported to PubMLST for gene annotation and species identification using *rplF* and ribosomal multilocus sequence typing (MLST) (40, 41). fHbp variant group/subfamilies were assigned to all unique alleles automatically upon submission to PubMLST. All allele numeric identifiers (IDs) used in this article are PubMLST designations.

The inability of these strains to grow on GC VCAT was assessed before confirmation of colistin sensitivity by disk diffusion using 25 µg colistin sulfate MAST disks (MAST, UK) on Mueller-Hinton agar (Oxoid, UK). A meningococcal control strain (“EMGM1”; C:2a:P1.5,2) was also tested. While disk diffusion is not the recommended method or validated for *Neisseria* spp., it served to provide confirmation that colistin is the agent against which these strains are susceptible (42).

### Historic MRU commensal panel.

A search of the PubMLST isolate database revealed 10 *Neisseria* commensal isolates that had been whole genome sequenced by the MRU in previous years. The presence of *lptA* and *fHbp* was determined using PubMLST before all strains were plated on GC VCAT to assess colistin tolerance. fHbp surface expression was also assessed.

### Analysis of UKMenCar4 carriage study strains.

To further investigate the impact of *lptA* on isolation using GC VCAT during carriage studies, all 1,675 UKMenCar4 carriage study genomes within the PubMLST were analyzed for the presence of *lptA* (4). Seventeen isolates lacking *lptA* were assessed for their ability to grow on GC VCAT.

### Assessing GC VCAT growth capability.

To determine the ability of strains to grow on GC VCAT, strains were plated on GC VCAT and incubated at 37°C with 5% CO_2_. Plates were inspected for growth and 24 and 48 h. Strains exhibiting growth at 24 h were considered able to grow on GC VCAT. Strains that exhibited microcolonies at 48 h were considered colistin sensitive; however, the presence of tolerant subpopulations is indicated in the Results. All strains were also grown on CBA in parallel to confirm viability of the culture.

### Measurement of fHbp surface expression using a modified meningococcal antigen surface expression assay (MEASURE) assay protocol.

Surface expression of fHbp was detected using flow cytometric analysis of the cells stained with the fHbp-specific monoclonal antibody MN86-994-11-1. The protocol used to prepare the fixed cells was a modification of the MEASURE protocol described by McNeil et al. (34). Strains were initially grown overnight at 37°C with 5% CO_2_ on GC II agar with Isovitalex (Becton Dickinson, United Kingdom). The standard MEASURE protocol involves incubation of the strains to the required concentration in liquid broth; however, many of the *Neisseria* commensal strains failed to grow during incubation in GC broth so liquid bacterial suspensions were prepared to the required final concentration (optical density at 650 nm of 0.5 to 0.55) directly from colonies on the overnight growth plate (no liquid incubation). The suspensions were centrifuged, resuspended in phosphate-buffered saline (PBS) solution containing 1% paraformaldehyde, and incubated at 4°C for 16 h. The fixed cells were then stained using an indirect antibody staining protocol with phycoerythrin (PE) as the fluorescent reporter as previously described (34). Flow cytometric analysis was performed on a BD FACSVia cytometer. Positive fHbp expression was defined as a mean fluorescence intensity (MFI) of at least 100 and at least three times the MFI of a nonspecific isotype control antibody stained under the same conditions. Within each assay run, two meningococcal strains (PMB1135 and PMB1745) were also tested as positive controls (34). To aid interpretation of the MFI read-outs, positive fHbp expression values were designated one of the following expression levels based on the fHbp-specific MFI to background signal ratio: low (≤5), medium (6 to 15), high (15 to 30), and very high (30+). These cutoffs were devised based on values observed in meningococci (34). As the background MFIs were reasonably consistent, the signal ratio correlated well with the fHbp-specific read-out (e.g., all strains designated “Low” had fHbp-specific read-outs of <1,000, and all strains designated “Very High” had read-outs of >10,000).

### fHbp nomenclature and sequence analysis.

The fHbp variant nomenclature used in this study, whereby unique fHbp gene and peptide variants are assigned arbitrary numeric identifiers, is curated within the PubMLST *Neisseria* database (40). Where appropriate, the fHbp variant groups (43) and/or subfamilies (44) to which the variants have been assigned (within PubMLST) are specified.

An assessment sequence similarity between the fHbp peptide variants found among commensal *Neisseria* and those observed among English and Welsh strains within the Meningitis Research Foundation’s Meningococcus Genome Library (MRF-MGL), was performed (45). At the time of the analysis, the MRF-MGL included all invasive English and Welsh invasive meningococcal strains isolated by Public Health England (PHE) Meningococcal Reference Unit (MRU) from July 2010 to September 2020 (*n* = 4,444). All unique fHbp peptide variant IDs possessed by these invasive strains (*n* = 254) were exported from PubMLST. To these, the five additional fHbp variants observed only among the commensal strains were added, and the peptide sequences for all these variants (total *n* = 259) were downloaded from PubMLST. In order to improve the accuracy of the analysis, for each peptide sequence, the leading Cys residue and variable Gly/Ser linker residues were removed so the first residue was Val/Ile (Val-8 in variant 1.1). The trimmed peptide sequences were then aligned using ClustalW in MEGA software version 4.0 using default parameters (46). A NeighborNet splits graph was generated from the aligned sequences using SplitsTree4 (47).

### Data availability.

The indexed genomes for all commensal isolates in this article can be accessed at https://pubmlst.org/organisms/neisseria-spp using the isolate IDs listed in Tables 1 to 4 (40). All numeric gene/peptide allele IDs are PubMLST designations, and the corresponding gene/peptide sequences can be freely downloaded at https://pubmlst.org/organisms/neisseria-spp.
